# Postoperative pain trajectories in total hip arthroplasty: an analysis of patient-reported outcomes

**DOI:** 10.1302/2633-1462.53.BJO-2023-0181.R1

**Published:** 2024-03-06

**Authors:** Kareem Omran, Daniel Waren, Ran Schwarzkopf

**Affiliations:** 1 Department of Public Health and Primary Care, University of Cambridge, Cambridge, UK; 2 Department of Orthopedic Surgery, NYU Langone Health, New York, New York, USA

**Keywords:** THA, pain outcomes, total hip artrhoplasty, growth mixture model, pain trajectory, osteoarthritis, post-operative recovery, PROMIS Intensity, Postoperative pain, Total hip arthroplasty (THA), Patient-Reported Outcomes Measurement Information System, BMI, preoperative pain, logistic regression model, hip disorders, retrospective cohort study, hip arthroplasty

## Abstract

**Aims:**

Total hip arthroplasty (THA) is a common procedure to address pain and enhance function in hip disorders such as osteoarthritis. Despite its success, postoperative patient recovery exhibits considerable heterogeneity. This study aimed to investigate whether patients follow distinct pain trajectories following THA and identify the patient characteristics linked to suboptimal trajectories.

**Methods:**

This retrospective cohort study analyzed THA patients at a large academic centre (NYU Langone Orthopedic Hospital, New York, USA) from January 2018 to January 2023, who completed the Patient-Reported Outcomes Measurement Information System (PROMIS) pain intensity questionnaires, collected preoperatively at one-, three-, six-, 12-, and 24-month follow-up times. Growth mixture modelling (GMM) was used to model the trajectories. Optimal model fit was determined by Bayesian information criterion (BIC), Vuong-Lo-Mendell-Rubin likelihood ratio test (VLMR-LRT), posterior probabilities, and entropy values. Association between trajectory groups and patient characteristics were measured by multinomial logistic regression using the three-step approach.

**Results:**

Among the 1,249 patients, a piecewise GMM model revealed three distinct pain trajectory groups: 56 patients (4.5%) in group 1; 1,144 patients (91.6%) in group 2; and 49 patients (3.9%) in group 3. Patients in group 2 experienced swift recovery post-THA and minimal preoperative pain. In contrast, groups 1 and 3 initiated with pronounced preoperative pain; however, only group 3 exhibited persistent long-term pain. Multinomial regression indicated African Americans were exceedingly likely to follow trajectory groups 1 (odds ratio (OR) 2.73) and 3 (OR 3.18). Additionally, odds of membership to group 3 increased by 12% for each BMI unit rise, by 19% for each added postoperative day, and by over four if discharged to rehabilitation services (OR 4.07).

**Conclusion:**

This study identified three distinct pain trajectories following THA, highlighting the role of individual patient factors in postoperative recovery. This emphasizes the importance of preoperatively addressing modifiable risk factors associated with suboptimal pain trajectories, particularly in at-risk patients.

Cite this article: *Bone Jt Open* 2024;5(3):174–183.

## Introduction

Total hip arthroplasty (THA) is a widely employed intervention for alleviating pain and improving function in patients with hip disorders, primarily osteoarthritis. While THA is generally a highly successful procedure, there remains significant variability in postoperative pain recovery.^1^ Studies indicate that about 23% of patients may encounter postoperative hip pain,^2-5^ with a subset even reporting stagnation or exacerbation of pain following the procedure.^4^ While loosening of implants, infections, periprosthetic fractures, and soft-tissue abnormalities are recognized causes of postoperative pain,^3^ numerous instances lack a clear correlation with radiological or mechanical irregularities.^1^

Factors that can influence THA outcomes involve both modifiable and non-modifiable risk factors. Modifiable risk factors can include preoperative level of symptoms, expectations, comorbidities, and mental state, as well as other socioeconomic variables.^2,6^ Conversely, non-modifiable risk factors include age, sex, and race.^7^ Identification of modifiable risk factors of increased postoperative pain is critical in guiding preoperative optimization. Meanwhile, understanding non-modifiable risk factors is useful in detecting at-risk patients, as well as determining surgical expectations, individualizing pain management, and guiding the informed decision-making process.

Patient-reported outcome measures (PROMs) are standardized, validated questionnaires that capture patients' perspectives on their health status.^8^ Patients undergoing THA often demonstrate significant enhancements in PROMs related to function and quality of life (QoL) within the first postoperative year; however, these improvements typically plateau in the following years.^9,10^ While this is well supported by scientific evidence, diverse recovery trajectories have been observed across distinct patient subgroups.^11-15^ Certain groups sustain long-term functional gains, whereas others exhibit short-to-medium term declines.^16^ Even among those achieving complete functional recovery, pain can persist, impacting QoL.^2^ Thus, pinpointing patient subsets potentially susceptible to persistent postoperative pain becomes crucial. Adopting such a targeted perspective fosters patient-centred discussions, setting individualized recovery benchmarks. This study aims to identify predictors of following a compromised postoperative pain recovery, aiming to refine preoperative counselling. The overarching goal is to guide at-risk patients towards health interventions that favour sustained pain relief benefits.

## Methods

### Study design and data collection

This retrospective cohort study used routinely collected patient data from a tertiary academic multicentre institute (NYU Langone Orthopedic Hospital, New York, USA). All patients who underwent a THA from January 2018 to January 2023 were included. Exclusion criteria were applied, with individuals being excluded if they had experienced any subsequent revision surgery; bilateral surgery; surgery involving multiple joints; or surgery indicated due to trauma. Data collected included patient characteristics and clinical details, such as sex, race, age, smoking status, BMI, postoperative length of stay (LOS), American Society of Anesthesiologists (ASA) grade, and discharge disposition.

As part of routine hospital policy, all patients were given Patient-Reported Outcomes Measurement Information System (PROMIS) intensity questionnaires during their visits to the clinic. Patients were further sent PROMIS intensity questionnaires digitally at one, three, six, 12, and 24 months postoperatively. As patient completion dates for these questionnaires did not always align with the projected intervals, the data was systematically grouped. Questionnaires completed from 180 days pre-surgery to surgery day were considered as baseline, 0 to 60 days post-surgery as one month, 61 to 120 days as three months, 121 to 240 days as six months, 241 to 545 days as one year, and 546 to 1,095 days as two years. Scores from these questionnaires were collected as calibrated T-scores, with a score of 50 representing the average for the USA general population, with higher scores indicating greater levels of pain. This study followed the STROBE reporting guidelines for observational studies.^17^

To address potential attrition and selection bias, patients were grouped into three categories: 1) responders were those who completed the baseline questionnaire and at least two follow-ups; 2) patients lost to follow-up included those who completed only the baseline and fewer than three total surveys; and 3) non-participants consisted of patients who opted out of the survey process altogether.

### Latent growth curve modelling

Growth mixture models (GMM) were used to investigate recovery trajectories post-THA using PROMIS scores and survey time intervals. To handle missing data during modelling, the full information maximum likelihood (FIML) algorithm was implemented, enabling estimation of trajectories for responders, regardless of if they did not complete surveys at all timepoints.^18,19^

### Identifying trajectory groups and model selection

To identify trajectories, a one-class solution was first estimated followed by iterative addition of classes up to a six-class solution. Alongside this, models with varying formulae for the slopes (linear, quadratic, or piecewise) were also considered.

Various fit indices, statistics, and classification diagnostics determined the best model. The Bayesian information criterion (BIC) and Akaike Information Criterion (AIC) were used, with lower values indicating better fit.^20^ The Vuong-Lo-Mendell-Rubin adjusted likelihood ratio test (VLMR-LRT) compared models with k classes to k-1 classes.^21^ A significant p-value (p < 0.05) indicated the model with k classes was superior. Classification diagnostics included average latent class posterior probabilities and entropy. Posterior probabilities close to 1.0, above 0.8, suggest accurate classifications.^22^ Entropy ranges from 0 to 1, with values over 0.8 indicating better class separation.^23^ Models with classes under 1% of the sample were rejected for stability and generalizability. Models were deemed successful if there was evidence of replication of the best log-likelihood, to avoid calculations based on local maxima, which lead to invalid solutions.^24^ Consequently, all models were run with 12,000 random starts.

As growth mixture models are sensitive to the assumption of normality,^25^ distribution of the scores were visualized, alongside calculation of skewness and kurtosis. Where the normality assumption failed, models were re-run using a Skewnormal and SkewT distributions on multiple dedicated 64-CPU virtual machines.^25^ However, these failed to converge, even with an increased 25,000 random starts, with the best log-likelihood unattainable. Thus, trajectory modelling resumed assuming normality.

Spaghetti plots were produced for the best fitting trajectories, which show latent classes overlayed on the patients’ individual trajectories. This enables visualization of whether the statistically generated trajectories are representative of the patient population. To ensure that the trajectories aligned with clinical plausibility, the best fitting model was subsequently evaluated by a clinical expert (RS).

### Association with trajectory group membership

To identify variables associated with sub-optimal trajectories, a three-step approach was used which adhered to the 'most likely class regression’ methodology.^26^ This began with modelling patient scores over time to identify latent trajectory groups using GMM. Participants were then assigned to a trajectory group based on their greatest posterior probability of membership. Finally, multinomial logistic regression was conducted with the allocated classes as outcome variables and patient characteristics as predictor variables.

### Ethical approval

Patient records and data were de-identified as part of our institutional quality improvement programme. Approval by our Institutional Review Board (IRB) was obtained prior to this study. Study protocols were maintained in accordance with the Declaration of Helsinki.^27^

### Statistical analysis

Data handling, manipulation, and regression models were conducted using RStudio version 4.2.2 (R Foundation for Statistical Computing, Austria). Mplus 8.8 (Mplus, USA) was used to conduct all GMM analyses.^24^

## Results

### Patient characteristics

**Fig. 1:**
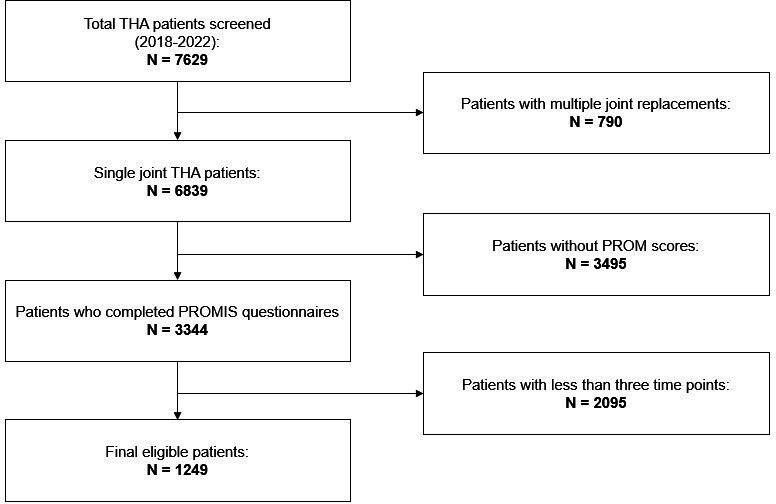
Flowchart illustrating the study population.

Out of 7,629 patients who underwent a THA procedure during the study period, 1,249 met the inclusion criteria (Figure 1). The typical patient had a median age of 66 years (interquartile range (IQR) 58 to 72) and a median BMI of 28.3 kg/m^2^ (IQR 24.7 to 32.5) upon presentation. In all, 62% of the participants were female. The median postoperative stay was one day (IQR 1 to 2). Notably, 277 patients (22.18% of the sample) had same-day discharge. Data completeness was high, with a 0% missing rate for most variables, except race (1.3%), smoking status (0.1%), discharge disposition (0.1%), and postoperative length of stay (7.3%). Table I details the patient characteristics and baseline demographics.

**Table I.** Baseline patient characteristics and PROMIS intensity scores (n = 1,249).

Characteristic	Data
Median age at surgery, yrs (IQR)	66 (58 to 72)
Median length of postoperative stay, days (IQR)	1.00 (1.00 to 2.00)
**Sex, n (%)**	
Female	780 (62)
Male	469 (38)
Median BMI, kg/m^2^ (IQR)	28.3 (24.7 to 32.5)
**Race, n (%)**	
White	940 (76)
African American	158 (13)
Asian	25 (2.0)
Other	110 (8.9)
**ASA grade, n (%)**	
ASA I to II	897 (72)
ASA III to V	352 (28)
**Smoking status, n (%)**	
Never smoker	656 (53)
Current smoker	62 (5.0)
Former smoker	530 (42)
**Discharge disposition, n (%)**	
Discharged from care services	1,179 (94)
Rehabilitation and therapy services	69 (5.5)
**Median PROMIS intensity, score (IQR)**	
Preoperative (baseline)	54 (51 to 59)
1 month postoperative	46 (43 to 51)
3 months postoperative	43 (38 to 50)
6 months postoperative	46 (40 to 51)
12 months postoperative	44 (40 to 51)
24 months postoperative	50 (43 to 53)

ASA, American Society of Anesthesiologists; IQR, interquartile range; PROMIS, Patient-Reported Outcomes Measurement Information System.

### Best fitting trajectory

**Fig. 2:**
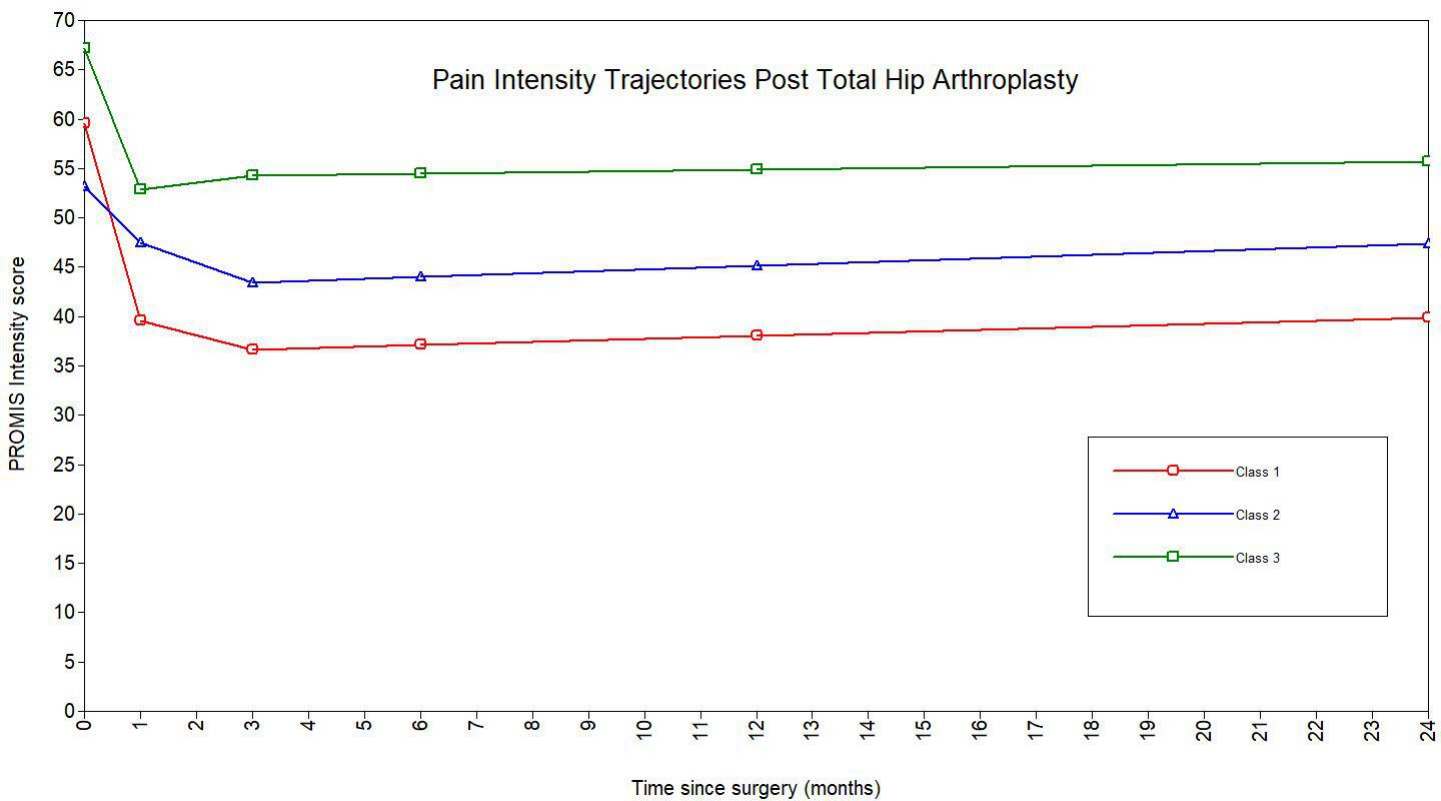
Piecewise three-class growth mixture model projecting latent trajectories in patient PROMIS intensity scores over the two-year postoperative period following total hip arthroplasty.

**Fig. 3:**
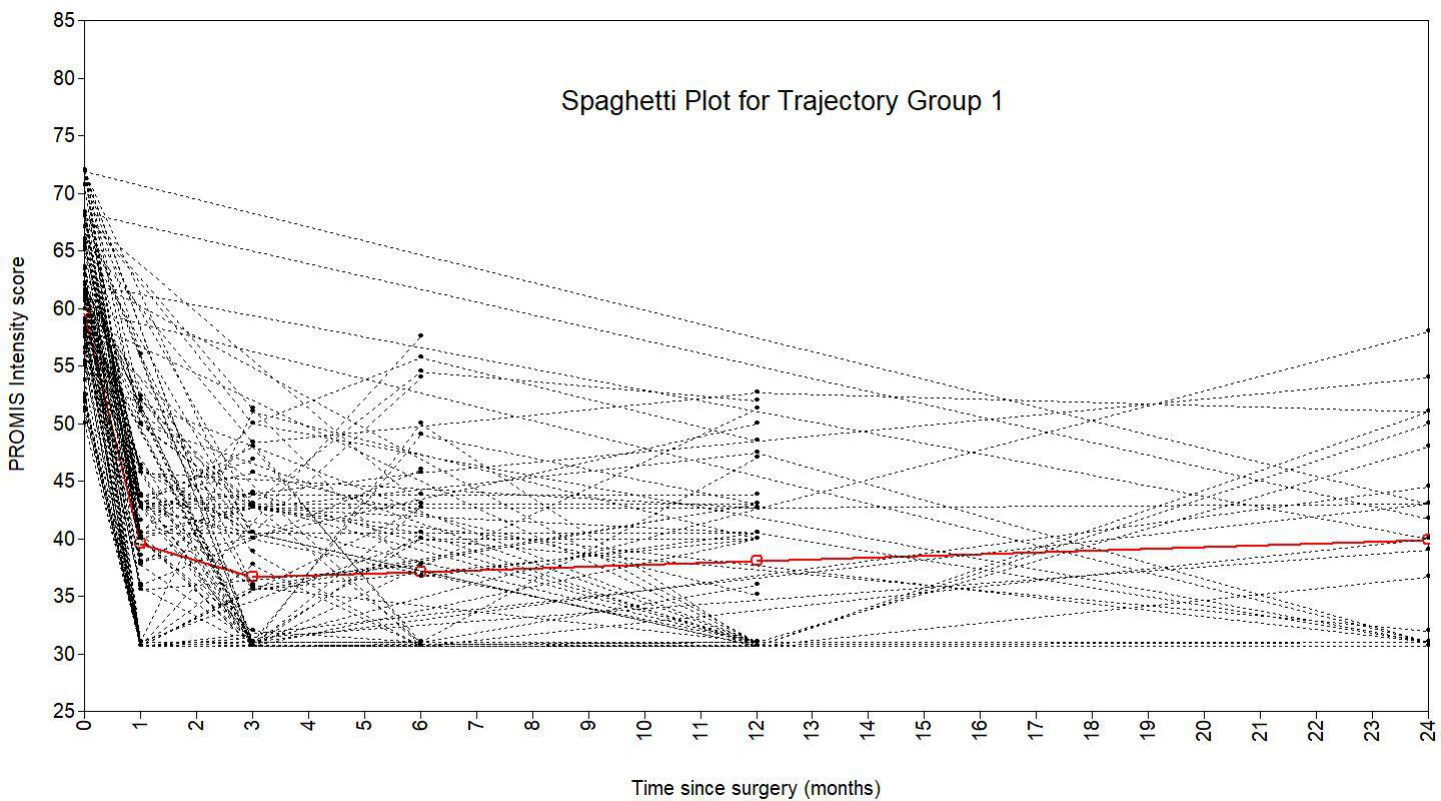
Spaghetti plot showing latent trajectory class 1, overlayed over the patients allocated to this trajectory, from the three-class piecewise PROMIS intensity growth mixture model.

**Fig. 4:**
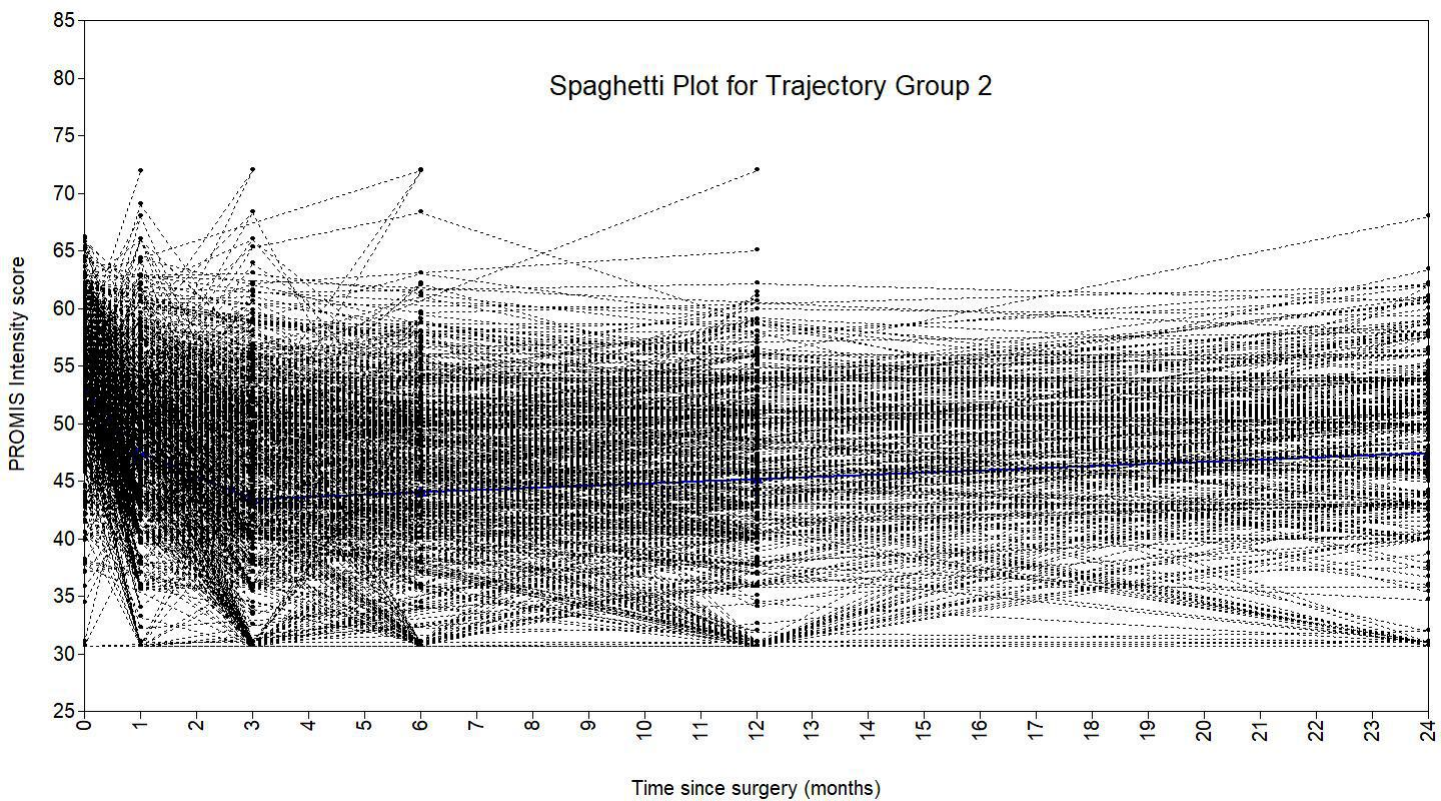
Spaghetti plot showing latent trajectory class 2, overlayed over the patients allocated to this trajectory, from the three-class piecewise PROMIS Intensity growth mixture model.

**Fig. 5:**
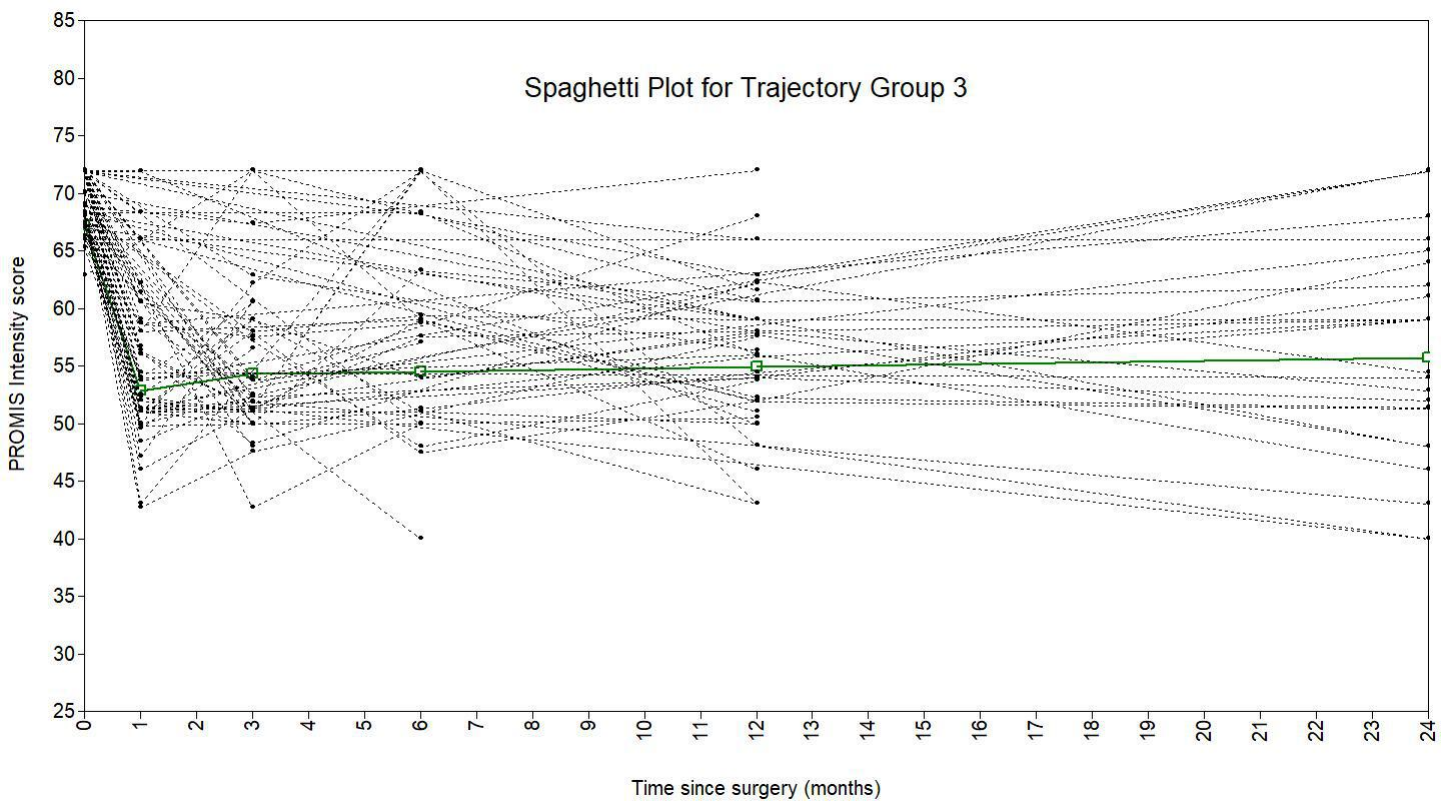
Spaghetti plot showing latent trajectory class 3, overlayed over the patients allocated to this trajectory, from the thre-class piecewise PROMIS intensity growth mixture model.

A piecewise growth mixture model with three trajectory classes best fit the data (Figure 2). This model had the lowest BIC value, a significant VLMR-LRT, an entropy of 0.85, a minimum diagonal posterior probability of 0.74, and a minimum class size of 3.9% of the sample (Table II). The model was described as having two ‘pieces’: baseline to one month, and one month to two years. There were 56 patients (4.5%) in group 1, 1,144 (91.6%) in group 2, and 49 (3.9%) in group 3. Group 2 showed rapid post-THA improvement and encompassed the majority of patients and was deemed the standard trajectory. Groups 2 and 3 began with higher preoperative pain, while group 2 recovered quickly within a month, and group 3 maintained high pain over two years. Spaghetti plots (Figures 3 to 5) showed good agreement for all classes.

**Table II.** Goodness of fit parameters of all growth mixture models generated for the PROMIS intensity pain questionnaire trajectories.

Model	Class	AIC	BIC	Entropy	Minimum PP	Minimum class size, %	VLMR-LRT
Linear	1	33110.41	33166.84	-	-	-	-
Quadratic	1	32758.94	32835.89	-	-	-	-
Piecewise (2 × 4)	1	31589.58	31671.662	-	-	-	-
Piecewise (3 × 3)	1	31888.62	31991.22	-	-	-	-
Linear	2	33088.21	33160.03	0.360	0.736	19.5	0.0032
Quadratic	2	32723.59	32821.07	0.878	0.755	3.0	0.0001
Piecewise (2 × 4)	2	31535.58	31643.31	0.849	0.804	4.3	0.0174
Piecewise (3 × 3)	2	31861.3	31989.55	0.795	0.753	6.5	0.1562
Linear	3	33075.22	33162.43	0.550	0.740	2.3	0.1167
Quadratic	3	32708.46	32826.45	0.623	0.705	3.6	0.0451
Piecewise (2 × 4)	3	31511.18	31644.56	0.846	0.737	3.9	0.0304
Piecewise (3 × 3)	3	31844.46	31998.37	0.584	0.706	4.5	0.2805
Linear	4	33073.38	33175.98	0.449	0.556	1.8	0.2479
Quadratic	4	32640.57	32779.08	0.603	0.688	4.8	0.1556
Piecewise (2 × 4)	4	31475.25	31634.28	0.742	0.746	3.4	0.0009
Piecewise (3 × 3)	4	31832.46	32012.01	0.646	0.702	2.2	0.5029
Linear	5	33072.64	33190.63	0.48	0.592	0.7	0.4958
Quadratic	5	32611.17	32770.2	0.637	0.677	3.0	0.118
Piecewise (2 × 4)	5	31432.9	31617.59	0.756	0.749	3.1	0.0631
Piecewise (3 × 3)	5	31813.65	32018.85	0.69	0.745	0.9	0.4137
Linear	6	33063.14	33196.52	0.519	0.596	0.5	0.0578
Quadratic	6	32582.66	32762.21	0.674	0.679	0.3	0.0003
Piecewise (2 × 4)	6	31422.39	31632.73	0.674	0.609	2.2	0.8159
Piecewise (3 × 3)	6	31804.2	32035.05	0.709	0.670	0.9	0.0671

2 x 4 refers to a model where baseline to one month is the first joint, whereas 3 x 3 refers to a model where baseline to three months is the first joint, with the rest of the time points being the second joint in both cases.

AIC, Akaike’s information criterion; BIC, Bayesian information criterion; PP, posterior probability; VLMR-LRT, Vuong-Lo-Mendell-Rubin likelihood ratio test.

### Association between allocated trajectory groups and patient characteristics

Multinomial logistic regression (Table III) revealed that African Americans had significantly higher odds of being in class 1 (odds ratio (OR) 2.73; 95% confidence interval (CI) 1.35 to 5.54) and class 3 (OR 3.18; 95% CI 1.46 to 6.93) compared to class 2. Furthermore, those in class 3 exhibited several distinct characteristics: they were more likely to have a higher BMI, with each unit increase in BMI raising the likelihood by 12% (OR 1.12; 95% CI 1.06 to 1.18). They also had extended postoperative stays, where each additional day raised the odds by 19% (OR 1.19; 95% CI 1.01 to 1.42). Additionally, individuals in class 3 had a notably higher odds of being discharged to rehabilitation and therapy services with an OR of 4.07 (95% CI 1.67 to 9.96) compared to those in class 2.

**Table III.** Results from the multinomial logistic regression model, indicating the odds ratio of allocation to trajectory classes 1 and 3 as opposed to the standard trajectory (class 2).

	Class 1			Class 3		
**Characteristic**	**OR**	**95% CI**	p-value*	**OR**	**95% CI**	p-value*
Age at surgery, yrs	1.01	0.98 to 1.04	0.600	1.03	0.99 to 1.07	0.110
Length of postoperative stay	1.04	0.84 to 1.29	0.700	1.19	1.01 to 1.42	0.041
**Sex**						
Female	-	-		-	-	
Male	0.77	0.42 to 1.42	0.400	0.87	0.44 to 1.72	0.7
BMI, kg/m^2^	1.00	0.96 to 1.06	0.900	1.12	1.06 to 1.18	< 0.001
**Race**						
White	-	-		-	-	
African American	2.73	1.35 to 5.54	0.005	3.18	1.46 to 6.93	0.004
Other	1.02	0.34 to 3.06	> 0.9	3.43	1.37 to 8.56	0.008
**ASA grade**						
I to II	-	-		-	-	
III to V	1.07	0.55 to 2.09	0.900	1.23	0.61 to 2.48	0.6
**Smoking status**						
Never smoker	-	-		-	-	
Current smoker	2.76	0.97 to 7.89	0.057	2.30	0.68 to 7.80	0.200
Former smoker	1.24	0.68 to 2.26	0.500	1.28	0.66 to 2.49	0.500
**Discharge disposition**						
Discharged from care services	-	-		-	-	
Rehabilitation and therapy services	1.59	0.49 to 5.16	0.400	4.07	1.67 to 9.96	0.002

* Multinomial logistic regression.

ASA, American Society of Anesthesiologists; CI, confidence interval; OR, odds ratio.

### Response analysis

At baseline, sex was the only significant difference between responders and patients lost to follow-up; 56% (566/1018) of non-responders were female, compared to 62% (780/1249) of responders (p < 0.001). In contrast, comparing participants to non-participants revealed differences in most baseline parameters, except age at surgery, which averaged 66 years for both. Non-participants were more often ethnic minorities (27% vs 23%; p = 0.003), had a higher ASA status of 3 to 5 (38% vs 29% of participants, p < 0.001), and 11% were discharged to rehabilitation (vs 5.7% of participants; p < 0.001). There were also fewer females among non-participants (56% vs 59%; p = 0.006), a higher average BMI (kg/m^2^; 28.7 vs 28.2 for participants; p = 0.018), and more smokers (9.4% vs 6.0% of participants; p < 0.001). The differences between categorical variables were assessed using Pearson's chi-squared test, while the Kruskal-Wallis rank sum test was used for continuous variables.

## Discussion

In this study, we discerned three unique pain recovery trajectories post-THA procedures and their associated clinical and demographic characteristics. The results were able to show that patients achieved most of their postoperative pain recovery by three months, and this was regardless of trajectory followed. This closely aligns with recovery patterns of patients undergoing THA in the literature.^28,29^ The majority of patients in our study (91.6%) exhibited typical and anticipated recovery patterns. However, through the use of GMM we were able to delineate the presence of two subgroups in our data, both characterized by high preoperative pain levels. These subgroups showed differentiation based on whether patients swiftly recuperated postoperatively or sustained elevated pain levels for the subsequent two years.

Those with prolonged pain showed alignment with prevailing views regarding the associations between the non-modifiable risk factors of pain such as ethnicity, as well as the detrimental effects of higher BMI on THA outcomes.^7,30,31^ However, this was the first study to assess these associations using longitudinal GMMs, with a focus on patient reported pain outcomes. Our study suggests that BMI's adverse impact on pain levels persists beyond just the preoperative and immediate postoperative phases and may have a long-term impact throughout the span of at least two years. This study also corroborates prior findings suggesting that African American patients might face an elevated risk of pain both pre- and post-THA.^32^ However, by identifying two distinct trajectories more commonly followed by African American patients, their divergence in long-term outcomes highlights that a high preoperative pain level does not inevitably equate to poorer postoperative outcomes. Such insights can be invaluable for these patients, reinforcing that while they may experience heightened preoperative pain relative to other ethnic cohorts, the postoperative advantages could be more pronounced. Furthermore, while ethnicity remains a non-modifiable risk factor for intensified pain, having an awareness of these increased odds can be beneficial for these patients, in both being able to set realistic recovery expectations, as well as help justify and motivate positive health behavioural changes for modifiable factors, such as BMI and reducing their long-term risk, given their already predisposed nature.

Further, a noteworthy observation from our study includes the four-fold increased odds of patients following a sub-optimal pain trajectory post-THA, if they were discharged to rehabilitation and therapy services. Based on institutional clinical insights, socioeconomic determinants emerge as potential contributors to this trend. Given the demographic and geographical distribution of our study cohort, a substantial portion of patients reside in multi-level structures, including high-rise buildings. Clinical consultations have highlighted a prevalent trend: patients inhabiting residences devoid of elevators or those living in solitude often demonstrate a preference for postoperative discharge to rehabilitation facilities. The underlying reason appears to be the lack of supportive infrastructure or adequate accessibility in their domiciliary settings, which might intensify their susceptibility and subsequently magnify their perception of postoperative pain.

This is significant as pain perception is not just a physical phenomenon; psychological elements play an indispensable role as well. This is exemplified in the concept of pain catastrophizing, where an exaggerated negative mental state, triggered by actual or anticipated painful experiences can amplify pain perception.^33^ The heightened fear or anxiety about pain can exacerbate its perception and can influence recovery trajectories. In the context of our findings, it is conceivable that the subset of patients discharged to rehabilitation and therapy services might be more prone to pain catastrophizing due to the compounded stress of socio-economic factors, and potentially, the lack of a supportive home environment. A study by Gonzalez et al^34^ also offers corroborative evidence, showing that post-THA patients discharged to rehabilitation facilities often report worse pain outcomes, a trend linked to lower educational levels and lower pre- and postoperative mental health scores. This finding aligns with our observations, highlighting the significant impact of mental health and socioeconomic factors on post-THA recovery trajectories, especially in the context of discharge disposition. Nonetheless, our analysis was constrained by the specific PROMs employed, and hence could not distinctly explore these interrelationships. Future research might benefit from incorporating insights from the Pain Catastrophizing Scale (PCS)^35^ to better quantify the impact of this phenomena.

### Strength and limitations

Our study’s methodology, characterized by its detailed longitudinal design, captures the complexities of pain trajectories post-THA with high levels of granularity. By incorporating six distinct evaluation points within the crucial two-year period following THA, we have provided an in-depth view of both short-term and intermediate recovery phases. This approach not only offers a comprehensive understanding of the pain recovery process, but also allows for a nuanced analysis of how factors, such as BMI, influence patient outcomes over time.

Crucially, the study’s focus extends beyond the immediate postoperative period, addressing a critical gap in existing research that has predominantly concentrated on broader outcomes like QoL and hip function. By zeroing in on the intricate patterns of pain recovery, particularly in the first three months post-surgery, our findings shed light on a pivotal phase of patient healing often overlooked in previous studies. This period, we discovered, is essential for significant postoperative recovery, underscoring the importance of early intervention and attentive care during these initial months.

In comparison to a study by Dainty et al,^36^ which explored pain trajectories over a longer span of five years in a larger cohort, our research provides a more focused lens on the early stages of recovery. Our findings align with Dainty et al’s^36^ conclusion that the state of the patient at six months is a key predictor of long-term outcomes, reinforcing the validity of our increased data granularity during this short postoperative period, and the relevance of our insights for understanding and improving long-term patient care post-THA.

However, there are limitations to consider. The observed sex discrepancy between study responders and those who discontinued participation raises potential concerns about attrition bias, which may challenge the external validity of our findings. While the sex distribution in this study is reminiscent of the wider THA patient population, suggesting some degree of representativeness, caution should still be applied when generalizing our findings.^37,38^ Additionally, the attrition rate in this study is comparable to others in the literature.^11,15,16^ Furthermore the possibility of selection bias is evident, given the underrepresentation of ethnic minorities and those with worse health statuses in our sample, pointing towards a potential barrier to participation among these groups. Since these patients mirror the characteristics of the patients following the sub-optimal trajectory, the implications of these findings may suggest that the proportion of patients who follow this trajectory may be underestimated. Alternatively, it may also be plausible that the patients who opted to participate did so due to their current status of having high preoperative pain, and hence the skewed and consistent association between ethnic minorities and higher preoperative pain. Regardless, while this study design was successful in capturing these trends, further investigations are needed to ascertain the causality and understand underlying mechanisms.

### Implications

In striving to offer holistic care to patients, clinicians must acknowledge the complex interplay of socio-economic and psychological factors when evaluating patients and planning postoperative care. While many derive immense relief from THA, a subset remains challenged by persistent pain. Thus, it is paramount for healthcare professionals to adopt a holistic approach, integrating both physical and mental health interventions, fine-tuned to individual patient characteristics and socio-economic backgrounds. Such an approach not only sets realistic recovery expectations, but also enriches preoperative consultations, enabling clinicians to provide tailored advice. The distinction of BMI as a significant, modifiable risk factor is especially notable. Preoperative counselling that provides actionable recommendations can nudge at-risk patients toward beneficial health behaviours, with the promise of the evidence-based potential advantages and reduced probability of prolonged postoperative pain.

**Take home message**

- This study highlights the need for a holistic approach in total hip arthroplasty, emphasizing the value of personalized preoperative counselling that considers socioeconomic, psychological, and health-related factors, such as BMI.

- Identifying predictors of postoperative pain recovery trajectories allows clinicians to set realistic expectations and provide tailored advice through actionable interventions.

- The discovery of distinct recovery paths for patients further calls for investigation into the underlying causes, emphasizing the potential for patient-tailored care.

## Data Availability

The data that support the findings for this study are available to other researchers from the corresponding author upon reasonable request.
